# Rewiring Dendritic Cell Immunity: The β-Catenin–TIM-3 Axis as a Target to Improve DC Cancer Vaccines

**DOI:** 10.3390/cancers18020201

**Published:** 2026-01-08

**Authors:** Chunmei Fu, Tianle Ma, Li Zhou, Qing-Sheng Mi, Aimin Jiang

**Affiliations:** 1 Center for Cutaneous Biology and Immunology, Department of Dermatology, Henry Ford Health, Detroit, MI 48202, USA; cfu1@hfhs.org (C.F.); lzhou1@hfhs.org (L.Z.); qmi1@hfhs.org (Q.-S.M.); 2 Immunology Program, Henry Ford Cancer Institute, Henry Ford Health, Detorit, MI 48202, USA; 3 College of Human Medicine, Michigan State University, East Lansing, MI 48824, USA; 4 Department of Computer Science and Engineering, School of Engineering and Computer Science, Oakland University, Rochester, MI 48309, USA; tianlema@oakland.edu; 5 Department of Internal Medicine, Henry Ford Health, Detroit, MI 48202, USA

**Keywords:** dendritic cell-based vaccines, β-catenin, TIM-3, immune checkpoint blockade, CD8 T cell immunity

## Abstract

The success of cancer vaccines relies on the ability of dendritic cells (DCs) to efficiently prime cytotoxic CD8 T cell responses against tumors. However, in solid tumors this process is often undermined by tumor-driven immunosuppression and intrinsic defects in DC activation. Among the signaling pathways implicated in DC dysfunction, β-catenin signaling has emerged as a key regulator of immune tolerance in DCs. In parallel, inhibitory receptors such as PD-L1 and TIM-3 on DCs have been recognized as critical DC-intrinsic brakes on CD8 T cell priming and on responses to immune checkpoint blockade (ICB). Recent work has identified a DC-intrinsic immunoregulatory circuit in which β-catenin activation in DCs—particularly in cross-presenting cDC1s—induces expression of TIM-3, thereby suppressing CD8 T cell cross-priming and limiting anti-tumor CD8 T cell immunity. This β-catenin–TIM-3 axis represents a previously underappreciated layer of negative regulation that may help explain, at least in part, the limited efficacy of many current DC-based cancer vaccines. In this review, we examine how β-catenin activation in DCs, particularly in cDC1s, induces TIM-3 and related inhibitory programs that suppress cross-priming of tumor antigen-specific CD8 T cells and constrain the efficacy of DC-based vaccines. We further discuss how selectively targeting this β-catenin–TIM-3 checkpoint axis—alone or together with PD-L1 and other β-catenin–linked receptors—could restore DC function and inform rational combinations of DC-based vaccination with ICB and other T cell-based immunotherapies.

## Simple Summary

Cancer vaccines rely on dendritic cells (DCs) to prime tumor-killing CD8 T cells, but tumors often develop mechanisms to suppress DC function. Emerging evidence shows that a signaling molecule called β-catenin in DCs induces the checkpoint receptor T cell immunoglobulin and mucin-domain containing-3 (TIM-3), which acts as a “brake” and reduces DCs’ ability to stimulate T cells. This review outlines how the β-catenin–TIM-3 axis undermines vaccine responses and highlights strategies to boost CD8 T cell immunity and improve combination therapies with immune checkpoint blockade (ICB).

## 1. Introduction

Dendritic cells (DCs) are widely recognized as the “engine” of the cancer–immunity cycle, a concept first articulated more than a decade ago by Chen and Mellman and more recently updated [1,2]. Across this cycle, DCs play distinct, non-redundant roles at both early and late stages. Initially, DCs capture and process tumor antigens, migrate to tumor-draining lymph nodes, and present peptide–MHC complexes together with co-stimulation and cytokines to prime de novo CD8 T cell responses, a process termed “cross-priming” (step 2–3). Later, DCs help maintain and restimulate effector T cells within tumors (step 5–7) to reinforce cytotoxic programs. Within this framework, DC-mediated cross-presentation of tumor antigens and the subsequent priming of tumor-specific naïve CD8 T cells in lymph nodes (steps 2–3), together with the intratumoral reactivation of effector T cells (steps 5–7), have emerged as critical, and often rate-limiting, bottlenecks in the generation of effective anti-tumor immunity [3,4].

As the so-called “professional” antigen-presenting cells (APCs), DCs are particularly efficient at acquiring cell-associated tumor antigens (for example, from apoptotic or necrotic tumor cells), cross-present these antigens onto MHC class I, and initiate de novo activation, clonal expansion, and functional differentiation of tumor-specific CD8 T cells capable of homing back to the tumor and mediating cytotoxic effector functions [3,5,6]. This specialized capacity for cross-presentation and CD8 T cell priming (cross-priming) has positioned DCs as an attractive platform for therapeutic vaccination. Clinical studies have shown that both ex vivo-generated and in vivo-targeted DC vaccines are safe and consistently induce tumor-specific T cell and antibody responses. Nevertheless, objective response rates in solid tumors have generally remained modest (~5–15%), indicating that DC vaccination alone is usually insufficient to overcome the multiple layers of tumor-induced immunosuppression [7,8,9,10]. Sipuleucel-T (Provenge), an autologous APC product enriched for DC-like cells pulsed ex vivo with a GM-CSF–prostatic acid phosphatase fusion protein, provides an important clinical proof-of-principle: in the phase III IMPACT trial, sipuleucel-T improved median overall survival by 4.1 months (25.8 vs. 21.7 months) and increased 3-year survival in metastatic castration-resistant prostate cancer, leading to the first FDA approval of a therapeutic cancer vaccine [11,12]. However, objective tumor regressions with sipuleucel-T are infrequent and most patients eventually progress [11,12], underscoring that DC-mediated cross-priming is necessary but not sufficient for durable clinical benefit and highlighting the need for next-generation DC-based vaccines and rational combinations with other immunotherapies.

A key reason for these limitations is that DC-based vaccines ultimately depend on host DCs to present antigens and initiate robust T cell responses [13,14,15]. However, tumors frequently impair DC development, survival, and function as a means of immune evasion. One major obstacle is tumor-mediated immunosuppression that targets DC function in cross-priming, thereby driving CD8 T cell tolerance (cross-tolerance) instead of effective immunity [3,5,16,17,18,19]. Within the TME, DCs often downregulate IL-12 and type I interferons, upregulate IL-10 and TGF-β, show reduced co-stimulation, and acquire a regulatory phenotype that blunts cross-priming and favors dysfunctional or exhausted T cells [3,16,17,18,19]. Together, these changes mean that the qualitative state of DCs within tumors and draining lymphoid organs—rather than antigen availability alone—is a major determinant of whether tumor-specific T cell responses are robust, durable, and clinically meaningful.

Despite the disappointing outcomes of many first-generation DC-vaccine trials, more recent neoantigen-based vaccines, including DC-based formats, have begun to show encouraging immunogenicity and clinical benefits [20,21,22,23,24]. Carreno et al. have reported on a clinical trial with neoantigen-loaded DCs [20], and several additional studies employing personalized neoantigen vaccines [21,22,23,24,25,26] have demonstrated strong T cell responses and preliminary evidence of clinical responses, suggesting that DC function can be harnessed effectively. Readers interested in a more comprehensive overview of cancer vaccine clinical trials are referred to recent in-depth reviews [27,28,29]. Consistent with this, recent findings that cDC1s (type 1 conventional DCs) play a critical role in cross-presenting tumor antigens for CD8 T cell priming and in determining the efficacy of other immunotherapies including immune checkpoint blockade (ICB) and adoptive cell transfer (ACT) [30,31,32,33,34], further support the idea that DC vaccines should not be abandoned but rather be refined either as a monotherapy or in combination with other immunotherapies. In this review, we examine how tumors reprogram DCs—particularly cDC1s—through pathways such as Wnt/β-catenin and DC-intrinsic checkpoint receptors, and we discuss strategies to restore DC function, in order to enhance CD8 T cell priming, reshape anti-tumor immunity, and improve the performance of current and emerging immunotherapies.

## 2. DC Subsets and the Vaccination Bottleneck

Although dendritic cells (DCs) are relatively rare, they comprise a heterogeneous family with distinct ontogeny and specialization [35]. Based on transcriptional programs, surface phenotype, and function, DCs can be broadly divided into type 1 and type 2 conventional/classical DCs (cDC1s and cDC2s), plasmacytoid DCs (pDCs), monocyte-derived DCs, and tissue-resident subsets such as Langerhans cells [7,8,36,37,38]. In the context of DC-based cancer vaccines, the central question is which of these subsets are responsible for cross-presentation of tumor antigens and cross-priming of CD8 T cells.

Notably, Batf3-dependent cDC1s have been shown to be critical for cross-presenting tumor antigens and eliciting anti-tumor CD8 cell responses. The clearest genetic evidence comes from two knockout mouse strains. Hildner et al. showed that deleting the transcription factor Batf3 eliminates the CD8a^+^/CD103^+^ cDC1s and abrogates cross-presentation, leading to impaired rejection of highly immunogenic tumors [39]. This work established Batf3-dependent cDC1s as an essential lineage for cytotoxic T cell immunity in vivo. Using Wdfy4^-/-^ mice, in which cDC1s are present but selectively defective in cross-presentation of cell-associated antigens, it was further shown that cDC1-mediated cross-presentation is required for tumor control as they fail to prime tumor-specific CD8 T cells or reject tumors [40]. Importantly, Batf3-dependent cDC1s can also contribute to anti-tumor immunity through mechanisms that are at least partly independent of cross-presentation [41].

Emerging evidence suggests that other DC subsets, particularly cDC2s, may acquire cross-presenting capabilities under specific inflammatory or tumor conditions [42,43,44,45,46], and that both pDCs and cDCs can cooperate to achieve optimal cross-priming and CD8 T cell immunity in some settings [47,48,49,50,51,52]. However, across multiple cancer models, the principal requirement for effective tumor antigen cross-presentation and CD8 T cell priming rests with cDC1s, which also play a major role in determining the efficacy of cancer immunotherapies including ICB and ACT.

**Figure 1:**
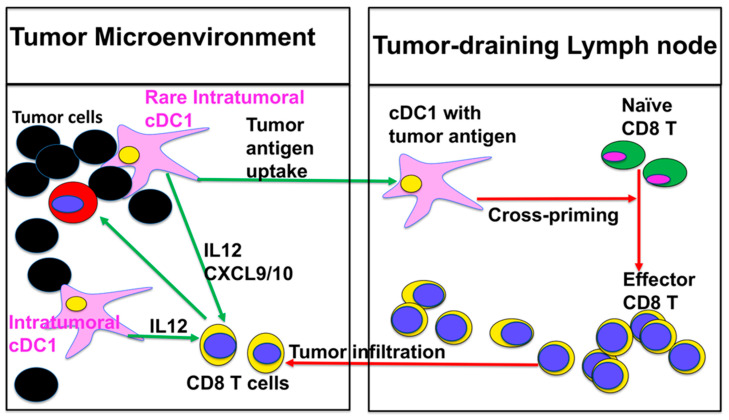
cDC1s as the central bottleneck of DC-based vaccines and T cell–based cancer immunotherapies. Rare intratumoral cDC1s capture tumor antigens, migrate to tumor-draining lymph nodes, and cross-present tumor antigens on MHC class I to prime naive tumor-specific CD8 T cells. Primed effector CD8 T cells then return to the tumor, where cDC1-derived IL-12s sustain effector function and support responses to immune checkpoint blockade and adoptive T cell therapy. Because cDC1s are few and often suppressed or excluded by tumors, the efficacy of DC-based vaccines and T cell-based immunotherapies largely depends on intact cDC1 function, making cDC1s both the engine and a key bottleneck of effective cancer immunotherapy.

Recent reports have further demonstrated that cDC1s not only transport tumor antigens to tumor-draining lymph nodes and cross-prime antigen-specific CD8 T cells but also recall (reactivate) antigen-experienced CD8 T cells in the tumor microenvironment (TME) (Figure 1). Broz et al. dissected the tumor myeloid compartment and identified intratumoral CD103^+^ cDC1s as a numerically tiny population that accounts for most of the T cell-stimulatory activity and is required for T cell-based immunotherapy [30]. In vivo imaging and single-cell analyses show that successful anti-PD-1 therapy requires tumor-infiltrated cDC1s that produce IL-12 to sustain T cell responses [53], and tumor-residing BATF3-dependent cDC1s are required for effector T cell trafficking and the efficacy of adoptive cell therapy (ACT) [30]. Salmon et al. showed that these CD103+ cDC1s are the only myeloid cells that carry intact tumor antigen to draining lymph nodes and prime tumor-specific CD8 T cells; expanding and activating them with Flt3L plus poly I:C markedly improved responses to PD-L1 and BRAF blockade [32]. Sánchez-Paulete and colleagues then demonstrated that Batf3-dependent cDC1s are essential for the efficacy of anti-PD-1 and anti-CD137 antibodies, directly linking cDC1-dependent cross-priming to checkpoint and co-stimulatory immunotherapy [33]. Spranger et al. showed that tumor-residing Batf3-dependent DCs are the dominant source of CXCL9/10 required for effector CD8 T-cell trafficking into tumors and for successful adoptive T cell therapy [34].

Taken together, these studies confirm the central role of cDC1s in generating and sustaining anti-tumor CD8 T cell immunity (Figure 1). However, cDC1s are rare, and tumors often target this lineage to inhibit its infiltration into the TME or to render the cells dysfunctional and/or suppressive, thereby impairing T cell activation and limiting the efficacy of DC-based vaccines and other immunotherapies [31,32,33,34]. A well-characterized example is provided by Spranger et al., who showed that WNT/β-catenin activation in melanoma cells suppressed CCL4, prevented recruitment of Batf3-lineage CD103 DCs into the tumor, blocked priming of tumor-specific CD8 T cells, and rendered tumors resistant to CTLA-4 and PD-1/PD-L1 blockade [31]. Thus, the rarity of cDC1s and their tumor-induced dysfunction create a major bottleneck for CD8 T cell priming and therapeutic efficacy (Figure 1). Understanding the pathways that govern the differentiation and function of DCs, particularly cDC1s, and developing strategies to restore their capacity to cross-prime tumor antigen-specific CD8 T cells will be critical not only for improving DC-based vaccines but also for maximizing the clinical benefit of ICB and ACT and addressing this vaccination bottleneck. Readers interested in a more comprehensive overview of DC subsets and their role in cross-presentation/cross-priming in the context of cancer immunity are referred to recent in-depth reviews [4,6,9,38,54,55,56].

## 3. β-Catenin Signaling in DCs

β-catenin, a central component of the canonical Wnt signaling pathway, was first implicated as a regulator of dendritic cell (DC) maturation and tolerance in studies examining E-cadherin-mediated adhesion and intestinal immunity [57,58]. We showed that disrupting E-cadherin–β-catenin interactions in DCs induces a distinct maturation program with regulatory features [57], whereas Manicassamy et al. demonstrated that activation of β-catenin in intestinal DCs skews responses toward tolerance, promoting regulatory T cell (Treg) induction and limiting inflammatory Th1/Th17 immunity [58]. These early studies established Wnt/β-catenin as a key intracellular switch that can bias DCs toward tolerance rather than immunity.

**Figure 2:**
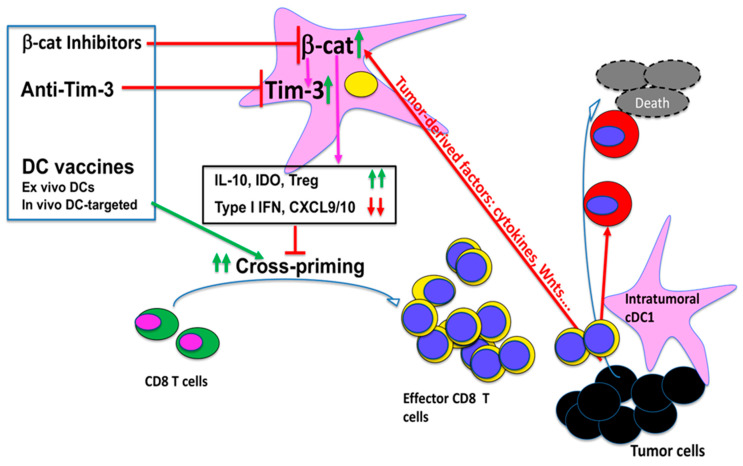
β-catenin–TIM-3 checkpoint axis in DCs limits cancer immunotherapy. Central role of β-catenin signaling in controlling DC-intrinsic checkpoint programs. Tumor-derived factors such as cytokines and Wnts activate β-catenin in DCs, driving a checkpoint program characterized by increased TIM-3 expression and elevated IL-10/IDO production. This β-catenin-driven state reduces cross-priming capacity and CD8 T cell responses. Therapeutic strategies can intervene either by DC-targeted modulation of β-catenin signaling or by combining DC vaccines with TIM-3 and potentially other inhibitory immune checkpoint blocking antibodies. Together, these approaches aim to invert the tolerogenic β-catenin–TIM-3 axis in DCs and restore productive priming and maintenance of anti-tumor CD8 T cell immunity.

Building on this foundation, work from our group and others has shown that tumors, including melanoma, induce the upregulation/activation of β-catenin in DCs, and β-catenin promotes the tolerogenic function of DCs to suppress anti-tumor CD8 T cell immunity [59,60,61,62,63] (Figure 2). Using a cDC1-targeted vaccine model, we have shown that tumors induce β-catenin in DCs to suppress cross-priming through an mTOR-IL-10-dependent pathway [59,60]. In that setting, activation of β-catenin in DCs inhibited cross-priming of CD8 T cells by upregulating mTOR-dependent IL-10, and blocking the β-catenin/mTOR/IL-10 pathway restored CD8 T cell immunity [60]. Similarly, Hong et al. showed that β-catenin activation in tumor-draining lymph node DCs promotes immune tolerance by inducing vitamin A-metabolizing enzymes and Treg differentiation, and that reducing β-catenin signaling in DCs enhances DC-mediated anti-tumor immunity and delays tumor growth [61].

Tumor-derived Wnt ligands provide an upstream mechanism for this tolerizing program (Figure 2). Holtzhausen and colleagues demonstrated that melanoma-derived Wnt5a upregulates durable IDO expression and activity in local DCs in a β-catenin-dependent manner, driving Treg expansion and immunotolerance [62]. In an autoimmune setting, Suryawanshi et al. reported that canonical Wnt ligand–LRP5/6–β-catenin signaling in DCs constrains Th1/Th17 differentiation while preserving regulatory T cell responses, thereby limiting experimental autoimmune encephalomyelitis and CNS pathology [64]. Zhao et al. further showed that paracrine Wnt5a–β-catenin signaling triggers a metabolic program in DCs characterized by increased fatty acid oxidation and oxidative phosphorylation, which enforces a tolerized DC state and contributes to immune evasion and resistance to immunotherapy [63].

However, β-catenin in DCs does not function solely as a brake. In our 2015 study, we found that β-catenin in DCs exerts opposite functions in different phases of the CD8^+^ T cell response: activation of β-catenin suppresses cross-priming via mTOR-dependent IL-10, but basal β-catenin activity is required to maintain primed CD8 T cells and memory responses [60]. These data highlight the context- and stage-dependent roles of β-catenin in DCs and suggest that complete blockade of this pathway may have complex effects on vaccine efficacy and long-term T cell immunity.

The mechanisms by which β-catenin promotes DC-mediated immunosuppression are therefore multifactorial and extend beyond IL-10 and metabolic reprogramming. Emerging evidence indicates that β-catenin signaling in DCs can also modulate inhibitory immune checkpoint pathways. Besides β-catenin, inhibitory immune checkpoint molecules such as PD-L1 and TIM-3 (T cell immunoglobulin and mucin-domain containing-3) are key players promoting the tolerogenic function of DCs, and their expression on DCs plays a critical role in determining the efficacy of anti-PD-1/PD-L1 and anti-TIM-3 immunotherapies [65,66,67]. In human DCs, fungi-induced Wnt/β-catenin activation has been shown to promote Treg responses at least in part by upregulating PD-L1, linking β-catenin signaling directly to checkpoint ligand expression on DCs [68]. We have recently shown that activation of β-catenin in DCs upregulates TIM-3, and TIM-3 blockade restores cross-priming in this β-catenin-active context [69,70]. Together with earlier work showing that PD-L1 and TIM-3 on DCs play a critical role in generating anti-tumor T cell immunity and responses to checkpoint blockade [65,67,71,72], these findings raise the possibility that β-catenin and checkpoint pathways cooperate within DCs to enforce tolerance. In the following sections, we therefore turn to inhibitory checkpoint molecules on DCs, their interaction with Wnt/β-catenin pathway, and how they might be targeted to enhance the efficacy of DC-based vaccines and other T cell-based immunotherapies.

## 4. DC-Intrinsic Checkpoint Pathways in Anti-Tumor Immunity: TIM-3, PD-L1, and Related Inhibitory Receptors

In parallel with Wnt/β-catenin signaling, inhibitory immune checkpoint molecules expressed by DCs themselves have emerged as critical regulators of T cell priming and the efficacy of immune checkpoint blockade (ICB). Among these, PD-L1 and TIM-3 constitute two of the best-defined DC-intrinsic inhibitory axes in cancer [65,67,71,72], with CTLA-4, LAG-3 and others providing additional context-dependent layers of regulation. Together, these molecules shape the magnitude and quality of anti-tumor T cell responses and influence how tumors respond to ICB therapies.

Early work by Brown et al. showed that human monocyte-derived DCs express both PD-L1 and PD-L2 and that antibody-mediated blockade of these ligands enhances T cell proliferation and cytokine production, particularly under suboptimal co-stimulation, identifying PD-1 ligands on DCs as active brakes on T cell activation [71]. This conceptual framework was extended by Peng et al., who demonstrated in murine tumor models that DC-specific deletion of PD-L1 attenuates T cell inhibition, enhances effector CD8 responses, and reshapes tumor control as well as responses to systemic anti–PD-L1 therapy, thereby placing PD-L1 on DCs at a key cellular node through which PD-1/PD-L1 blockade acts [72].

Two 2020 studies then firmly positioned PD-L1^+^ DCs at the center of PD-1/PD-L1 ICB biology. Oh et al. used conditional genetics and mixed bone marrow chimeras to demonstrate that PD-L1 expression by DCs is essential for effective priming and maintenance of anti-tumor CD8 T cell responses [65]. Selective loss of PD-L1 in DCs, but not in tumor cells or macrophages, profoundly altered tumor control and the efficacy of PD-1/PD-L1 blockade, indicating that DCs are a dominant site of action for these antibodies. In parallel, Mayoux et al. independently showed that DCs dictate responses to PD-L1 blockade in both preclinical models and patients: PD-L1 is highly expressed on peripheral and tumor-associated DCs, and anti–PD-L1 antibodies disrupt PD-L1–B7-1 cis interactions on DCs, thereby freeing B7-1 to engage CD28 and enhance co-stimulation [66]. A DC-enriched transcriptional signature, including PD-L1^+^ DCs, correlated with clinical benefit to atezolizumab (anti-PD-L1) in renal cell carcinoma and non-small cell lung cancer, underscoring the functional importance of DC PD-L1 in human ICB responses [66].

In parallel to PD-L1, TIM-3 has emerged as another important DC-intrinsic checkpoint axis. Recent studies have identified that TIM-3 (T cell immunoglobulin and mucin-domain containing-3), a checkpoint receptor originally described on IFN-γ-producing T cells [73], as being highly expressed on tumor-associated DCs, particularly cDC1s [74,75,76]. In this context, TIM-3 functions not simply as a T cell exhaustion marker but as a DC-resident inhibitory receptor that constrains innate sensing, chemokine production, and cross-priming. De Mingo Pulido et al. showed that CD103^+^ DCs (cDC1s) in breast tumors express high levels of TIM-3 and that this receptor regulates their contribution to chemotherapy responses [74]. In paclitaxel-treated breast cancer models, TIM-3 blockade enhanced tumor control in a CD103^+^ DC–dependent manner. Mechanistically, TIM-3 inhibition increased CXCL9 (and to a lesser extent CXCL10) production by cDC1s, improved CD8 T cell recruitment and effector differentiation, and augmented the therapeutic benefit of chemotherapy [74].

Building on this work, de Mingo Pulido et al. subsequently demonstrated that TIM-3 also limits activation of the cGAS–STING pathway in intra-tumoral cDC1s by suppressing uptake of extracellular DNA, thereby restraining type I IFN–dependent CXCL9 induction and the efficacy of TIM-3 blockade combined with paclitaxel chemotherapy [77]. In this study, loss or blockade of TIM-3 increased endocytic uptake and cytoplasmic localization of tumor-derived DNA in XCR1^+^ cDC1s, with downstream activation of cGAS–STING signaling; genetic deletion of Cgas or Sting, or disruption of HMGB1–DNA binding and galectin-9-induced TIM-3 clustering, impaired both chemokine production and the therapeutic synergy between TIM-3 blockade and chemotherapy [77]. Human peripheral blood cDC1s similarly increased extracellular DNA uptake upon TIM-3 blockade, indicating that this DC-intrinsic TIM-3–cGAS–STING axis is conserved across species.

Dixon et al. further clarified the locus of TIM-3 function using conditional TIM-3 deletion [67]. Loss of TIM-3 in DCs—but not in macrophages or CD4/CD8 T cells—was sufficient to unleash robust anti-tumor immunity and sensitize tumors to TIM-3 blockade. TIM-3-deficient DCs showed augmented inflammasome activation, with increased reactive oxygen species, NLRP3 activation, and IL-1β/IL-18 production; pharmacologic inhibition of inflammasome signaling abrogated these benefits [61]. Taken together with the de Mingo Pulido studies, these data position TIM-3 as a central DC-intrinsic hub that simultaneously restrains cGAS–STING–dependent type I IFN and chemokine production and inflammasome activation, thereby limiting anti-tumor CD8 T cell responses. Together with the PD-L1 studies, these findings support a model in which PD-L1 and TIM-3 on DCs represent two checkpoint axes that critically shape anti-tumor CD8 T cell priming and mediate the in vivo effects of PD-1/PD-L1- and TIM-3-targeted therapies.

Additional DC-expressed inhibitory receptors, such as CTLA-4 and LAG-3, reinforce the broader concept of DC-intrinsic checkpoint wiring. CTLA-4 is inducibly expressed on human monocyte-derived DCs and is upregulated upon maturation; its cross-linking inhibits DC maturation and antigen presentation and enhances regulatory features, including IL-10 and IDO expression [78]. LAG-3 is shown to express on plasmacytoid DCs (pDCs), where it regulates their homeostasis and contributes to their immunoregulatory function [79]. Although the function of these molecules on DCs are less studied than PD-L1 and TIM-3, they highlight that DC-intrinsic checkpoint programs can actively determine priming strength and quality. Moreover, DC subset-focused profiling now shows that PD-L1 and TIM-3 are differentially expressed across cDC1, cDC2 and pDC populations in both health and cancer, emphasizing that each subset brings a distinct checkpoint “fingerprint” to anti-tumor immunity [76].

Collectively, these data position PD-L1^+^ and TIM-3^+^ DCs—as well as DCs expressing CTLA-4, LAG-3 and related receptors—as central gatekeepers of cross-priming and ICB responses, and they raise an important mechanistic question: which upstream pathways coordinate the induction of these inhibitory programs on DCs? Emerging evidence points to Wnt/β-catenin as a key integrator of DC-intrinsic checkpoint expression, as discussed in Section 5.

## 5. β-Catenin–Integrated DC Checkpoint Programs: The β-Catenin–TIM-3 Axis and Related Pathways

Our own work integrates TIM-3 into the β-catenin framework described above. In a cDC1-targeted vaccination model, activation of β-catenin in DCs upregulates TIM-3, suppresses cross-priming, and impairs vaccine-induced CD8 T cell responses; TIM-3 blockade restores cross-priming in this β-catenin-active context [69]. In this paper, we showed that β-catenin activation in DCs (i) upregulates TIM-3 expression on cDC1s, (ii) reduces the frequency and IFN-γ production of gp100-specific (Pmel-1) CD8 T cells at priming and recall, and (iii) reprograms vaccine-primed CD8 T cells towards diminished effector and memory transcriptional signatures, as revealed by single-cell RNA sequencing.

Consistent with the notion that anti-TIM-3 enhances DC function, TIM-3 blockade in CD11c-β-catenin^active^ mice—which model tumor-induced β-catenin-mediated DC dysfunction [59]—restores cross-priming induced by cDC1 (DEC-205)-targeted vaccination and, when combined with DEC-205-targeted vaccines, further improves anti-tumor efficacy [69]. Functionally, anti-TIM-3 treatment in this setting rescued both primary and memory gp100-specific CD8 T cell responses to levels comparable to wild-type mice and, in B16F10-bearing hosts, significantly slowed tumor growth and reduced tumor mass when given together with DC-targeted vaccination, whereas TIM-3 blockade alone had minimal impact on tumor control [69]. Together, these data define a β-catenin–TIM-3 axis in DCs as a mechanistic brake on DC vaccine-induced CD8 T cell immunity and demonstrate that selectively targeting this axis can enhance anti-tumor efficacy of DC-based vaccines (Figure 2).

Notably, TIM-3 is also upregulated in tumor-associated cDC2s across several tumor models [75,80], suggesting broader induction across DC subsets. In support of this, RNA-sequencing of DCs with active β-catenin (from CD11c-β-catenin^active^ mice) reveals that β-catenin upregulates *Havcr2* (gene for TIM-3) across multiple DC subsets, including cDC2 populations, in addition to cDC1s [70], indicating that β-catenin can coordinate a DC-wide checkpoint program rather than acting on cDC1s alone. Additional work in human systems, including the expression profiling by Carenza et al. and functional studies showing that TIM-3 signaling can shape type 2 conventional DC (cDC2) responses in infection and STING agonist settings [76,81], supports the idea that TIM-3-high cDC2s could also be harnessed or reprogrammed in cancer to amplify vaccine-induced CD4^+^ and helper-dependent CD8 responses.

Together with the broader literature on PD-L1, CTLA-4, and LAG-3 in DCs, these data support a working model in which β-catenin collaborates with DC-resident checkpoints—PD-L1, TIM-3 and, in specific contexts, CTLA-4 and LAG-3—to install a tolerogenic DC state that restricts the magnitude and quality of CD8 T cell priming. While our data only showed that β-catenin upregulates TIM-3 and PD-L2 among these checkpoint molecules [70], a recent study by Karnam et al. in human monocyte-derived DCs demonstrates that β-catenin signaling can induce PD-L1 expression, IL-10 production, and Treg differentiation in response to fungal stimuli, and that β-catenin inhibition reduces PD-L1 and selectively attenuates Treg induction while preserving Th1 responses [68]. These findings are consistent with our murine data and support the concept that β-catenin promotes a PD-L1^+^/TIM-3^+^ tolerogenic DC phenotype across species.

Importantly, *CTLA-4* itself is a direct transcriptional target of Wnt/β-catenin signaling in melanoma cells: Shah et al. demonstrated that Wnt-3a activation increases *CTLA-4* mRNA and protein and that a TCF/LEF-binding site in the *CTLA-4* promoter is required for β-catenin responsiveness [82]. Although this work was performed in tumor cells rather than DCs, it provides a proof-of-principle that *CTLA-4* lies within the canonical β-catenin target gene repertoire and raises the possibility that, in specific microenvironments, β-catenin could also modulate CTLA-4 expression in DCs. In the same line, LAG-3 and PD-1 are tightly regulated by the β-catenin upstream kinase GSK-3 in T cells: small-molecule GSK-3 inhibition increases T-bet, which in turn represses *Pdcd1* (PD-1) and *Lag3* (LAG-3) transcription and synergizes with anti–LAG-3 or anti–PD-1 to enhance tumor control [83,84,85]. Taken together, these observations extend β-catenin control of immune checkpoints beyond TIM-3 and PD-L1, implicating other checkpoint receptors including CTLA-4 and LAG-3 in a broader “β-catenin–checkpoint axis” that operates across multiple immune and tumor cell types.

Within DCs, these studies support a working model in which PD-L1 and TIM-3 on DCs, together with potentially CTLA-4 and LAG-3 under certain circumstances (see above), and other receptors, provide additional context-dependent layers of regulation. In this setting, β-catenin signaling emerges as an upstream integrator that can drive both PD-L1 and TIM-3 expression and enforce a tolerogenic DC state. The 2024 Vaccines data provide direct functional validation of this concept: combining a cDC1-targeted DEC-205hgp100 vaccine with TIM-3 blockade overcame β-catenin-mediated DC dysfunction and yielded superior control of B16F10 melanoma compared with vaccination alone [69]. These findings argue that selectively targeting β-catenin-driven, DC-intrinsic checkpoints (starting with the β-catenin–TIM-3 axis and potentially extending to PD-L1, CTLA-4 and LAG-3 where they are β-catenin-responsive) could substantially boost the potency and breadth of DC vaccines.

In future applications, this β-catenin–checkpoint axis could be leveraged in at least two ways: (i) rational combinations of DC-targeted vaccines with anti-TIM-3, anti-PD-L1, or other checkpoint antibodies, delivered in schedules optimized to coincide with DC priming; and (ii) pharmacologic or genetic modulation of β-catenin in defined DC subsets (e.g., cDC1 and TIM-3-high cDC2) to prevent installation of a tolerogenic checkpoint program during vaccination. Embedding these strategies into current DC vaccine platforms has the potential to convert tolerogenic, checkpoint-rich DCs into potent inducers of durable anti-tumor CD8 and CD4 T cell immunity.

## 6. Discussion and Perspectives: Positioning the β-Catenin–TIM-3 Axis in DC-Centered Cancer Vaccines

While we use CD11c-β-catenin^active^ mouse to uncover the β-catenin–TIM-3 axis in DCs, it should be noted that we and others have shown that multiple tumors induce up-regulation and activation of β-catenin in DCs, including tumor-associated DCs, to suppress anti-tumor CD8 T cell responses [59,60,61,62,63]. Together with evidence that tumor-associated DCs (TADCs), including both cDC1s and cDC2s, express higher levels of TIM-3 [74,75,76,86], these observations support the idea that the β-catenin–TIM-3 axis in DCs is an important mechanism by which tumors suppress anti-tumor immunity. Supporting this idea, we found that β-catenin upregulates *Havcr2* (gene for TIM-3) in DCs and that anti-TIM-3 treatment completely restores cross-priming in CD11c-β-catenin^active^ mice [69], identifying TIM-3 as a tractable target for modulating DC-based vaccination responses. These effects occur without major changes in TIM-3, CTLA-4, PD-1, or Lag-3 expression on primed antigen-specific CD8 T cells, consistent with a predominantly DC-intrinsic mechanism. Together, these data support a β-catenin–TIM-3 axis—and more broadly a β-catenin–checkpoint axis—as an important regulator of DC function in immunity versus tolerance.

TIM-3 is not simply a marker of exhausted T cells but a component of an integrated, β-catenin-tuned checkpoint program in DCs, with PD-L1 and possibly CTLA-4, LAG-3 and other receptors providing additional context-dependent inhibitory layers. Given that PD-L1 has already been shown to be regulated by β-catenin in human DCs [68], and *CTLA-4* and Lag-3 either as a direct β-catenin target or regulated by a component of the β-catenin signaling pathway (for example, GSK-3) [82,83], it is plausible that different tumors engage distinct sets of checkpoint receptors to dampen anti-tumor immunity. This suggests that effective therapeutic combinations should consider DCs—in addition to T cells—as primary pharmacologic targets. For vaccines, that translates into three design principles: (i) deliver antigen plus strong innate cues to cDC1s (and, where available, TIM-3-high cDC2s); (ii) transiently inhibit TIM-3, PD-L1 and related DC-intrinsic checkpoints at the priming site; and (iii) then combine systemic ICB therapy to sustain and broaden T cell responses. Because tumor-induced β-catenin activation can raise checkpoint expression across multiple DC clusters, these strategies could be particularly relevant for tumors with sparse or dysfunctional cDC1s. For broader discussion of key translational hurdles that continue to limit DC vaccination—particularly tumor microenvironment constraints, antigen selection, and tumor-driven DC dysfunction—we refer readers to several recent in-depth reviews [9,29,87,88,89].

At the same time, the organization of these signaling pathways suggests caution when considering overly broad interventions. For example, the β-catenin upstream kinase GSK-3 has divergent roles in DCs and T cells in a context-dependent manner;s for instance, our recent report showed that deletion of GSK-3β does not lead to activation of β-catenin in DCs [70]. An important translational goal is to decouple DC-focused β-catenin/TIM-3 modulation from global Wnt or GSK-3 targeting, using route, timing, and cell-specific delivery to restrict the window of action to DC priming. In this setting, single-cell and spatial transcriptomic readouts of β-catenin activity, TIM-3 and PD-L1 expression, and cGAS–STING engagement in DC subsets may serve as both mechanistic biomarkers and tools for patient stratification. For clinical translation, a central question is how to combine existing agents in a mechanistically rational way. Vaccine platforms that deliver antigens to cDC1s, incorporate potent innate agonists, TIM-3 and PD-1 axis antagonists, and—eventually—more selective β-catenin modulators can be assembled into DC-centric regimens that explicitly account for the β-catenin–TIM-3 checkpoint state in DCs. Conceptually, DC-targeted, systematic modulation of DC-intrinsic checkpoints and β-catenin signaling provides a framework for designing next-generation cancer vaccines and checkpoint blockade strategies that aim to enhance the quality and durability of anti-tumor immune responses.

## 7. Conclusions

DCs—particularly the rare Batf3-dependent cDC1 subset—represent a major bottleneck in the cancer–immunity cycle (Figure 1), and tumors exploit pathways such as Wnt/β-catenin and DC-intrinsic checkpoints (notably TIM-3 and PD-L1) to blunt cross-priming and limit the efficacy of cancer immunotherapies (Figure 2). Accordingly, next-generation DC-based vaccines will likely require rational, temporally coordinated combinations that deliver antigens plus strong cDC1-targeted activation while relieving the β-catenin-linked checkpoint “brake” during priming, thereby driving stronger and more durable CD8 T cell responses and improving outcomes with ICB.

## Data Availability

Not applicable.

## Abbreviations

The following abbreviations are used in this manuscript:

DCs	dendritic cells
APCs	antigen-presenting cells
ICB	immune checkpoint blockade
MoDCs	monocyte-derived DCs
BMDCs	bone marrow-derived DCs
GSK-3	glycogen synthase kinase-3
IDO	indoleamine 2,3-dioxygenase
